# Comprehensive analysis of *CRP, CFH Y402H* and environmental risk factors on risk of neovascular age-related macular degeneration

**Published:** 2008-08-11

**Authors:** Ivana K. Kim, Fei Ji, Margaux A. Morrison, Scott Adams, Qingrun Zhang, Anne Marie Lane, Antonio Capone, Thaddeus P. Dryja, Jurg Ott, Joan W. Miller, Margaret M. DeAngelis

**Affiliations:** 1 Department of Ophthalmology, Harvard Medical School, Massachusetts Eye and Ear Infirmary, Boston, MA; 2 The Laboratory of Statistical Genetics, Rockefeller University, New York, NY; 3 Beijing Institute of Genomics, Chinese Academy of Sciences, Beijing, China; 4 Associated Retinal Consultants, P.C., William Beaumont Hospital, Royal Oak, MI

## Abstract

**Purpose:**

To examine if the gene encoding C-reactive protein (*CRP*), a biomarker of inflammation, confers risk for neovascular age-related macular degeneration (AMD) in the presence of other modifiers of inflammation, including body mass index (BMI), diabetes, smoking, and complement factor H (*CFH*) Y402 genotype. Additionally we examined the degree to which *CRP* common variation was in linkage disequilibrium (LD) within our cohort.

**Methods:**

We ascertained 244 individuals from 104 families where at least one member had neovascular AMD, and a sibling had normal maculae and was past the age of the index patient’s diagnosis of neovascular AMD. We employed a direct sequencing approach to analyze the 5′-promoter region as well as the entire coding region and the 3′-untranslated region of the *CRP* gene. *CFH* Y402 genotype data was available for all participants. Lifestyle and medical factors were obtained via administration of a standardized questionnaire. The family-based association test, haplotype analysis, McNemar’s test, and conditional logistic regression were used to determine significant associations and interactions. Haploview was used to calculate the degree of LD (*r*^2^) between all *CRP* variants identified.

**Results:**

Six single nucleotide polymorphisms (SNPs; rs3091244, rs1417938, rs1800947, rs1130864, rs1205, and rs3093068) comprised one haplotype block of which only rs1130864 and rs1417938 were in high LD (*r*^2^=0.94). SNP rs3093068 was in LD but less so with rs3093059 (*r*^2^=0.83), which is not part of the haplotype block. Six SNPs made up six different haplotypes with ≥ 5% frequency, none of which were significantly associated with AMD risk. No statistically significant association was detected between any of the nine common variants in *CRP* and neovascular AMD when considering disease status alone or when controlling for smoking exposure, BMI, diabetes, or *CFH* genotype. Significant interactions were not found between *CRP* genotypes and any of the risk factors studied. No novel *CRP* variation was identified.

**Conclusions:**

We provide evidence that if elevated serum/plasma levels of *CRP* are associated with neovascular AMD, it is likely not due to genetic variation within *CRP*, but likely due to variations in some other genetic as well as epidemiological factors.

## Introduction

The advanced stages of age-related macular degeneration (AMD) are responsible for the majority of visual loss observed in the developed world. In the United States, about 1.75 million people over the age of 50 years have advanced AMD, mostly in the form of neovascular AMD, in at least one eye, and it is predicted that this number will increase to 2.95 million individuals by 2020 [1]. The initial or acute phase response of the immune system to infection or other stressors involves the release of cytokines such as C-reactive protein (*CRP*) [2,3]. Measurement of such inflammatory markers in serum or plasma has been shown to predict risk of advanced forms of AMD [4,5], lending support to the hypothesis that AMD may be in part be a chronic inflammatory systemic disease. However, prospective studies from the Cardiovascular Health Study and Beaver Dam Eye Study concluded that circulating levels of *CRP* were not associated with either early or advanced AMD [6,7]. Identifying biomarkers that may predict risk of the more advanced stages of AMD may point to pharmacological targets relevant to preventing or delaying progression of disease. Therefore it is important that it be definitively determined if CRP is a valuable biomarker or prognostic tool for AMD risk. Evidence for the role of inflammation in AMD also comes from genetic studies showing that the most consistently reported genetic risk factor for both early and advanced forms of AMD is the Y402H disease-associated variant in the complement factor H gene (*CFH*) [8-12]. Moreover, this disease-associated variant is located in a binding site for *CRP*, and serum from AMD patients homozygous for *CFH* 402H were shown to have decreased binding to the *CRP* protein [13].

It is well established that common genetic variation within *CRP* are encompassed by seven single nucleotide polymorphisms (SNPs) that have been associated with circulating *CRP* levels [14-21], but it is unclear whether these common variations in CRP are associated with AMD risk. Lack of agreement exists between the two studies conducted to date on *CRP* variation and AMD risk. Specifically, data from the Netherlands demonstrated that *CRP* haplotypes associated with higher circulating *CRP* levels increase or decrease AMD risk depending on an individual’s *CFH* Y402H genotype [22]. However, data from the Physicians Health Study did not find an association between common genetic variation in *CRP* and risk of AMD even when controlling for *CFH* 402H genotype [23]. Common variation was defined differently between these two studies and may partly explain the difference in findings

Therefore, we employed a direct sequencing approach to encompass both sets of SNPs previously evaluated for their association with AMD risk [22,23] and also uncover any novel variation that could be associated with AMD risk within the *CRP* gene. Our study design also included controlling for factors that could modify *CRP* expression as well as risk of AMD, including *CFH* genotype, smoking, body mass index (BMI), and diabetes, reducing the likelihood of observing false positive correlations. Our study population consisted of 244 individuals from 104 families [1,24]. The affected or index patient was in the upper 10% of disease severity and the other member, the unaffected sibling, was in the bottom 10%–30% of disease severity (AREDS category one or less). We have previously demonstrated that such types of sib pairs can be powerful in identifying the contribution that many genetic variants, even those with a modest effect, along with smoking make simultaneously to AMD susceptibility [25,26].

Mathematical analyses indicate that the evaluation of sib pairs who are extremely discordant for a multifactorial trait can be the most informative for identifying the genetic variants that govern the trait and may be 40 times more powerful than case-controls study designs [27,28].

## Methods

### Patient population

The protocol was reviewed and approved by the Institutional Review Boards at the Massachusetts Eye and Ear Infirmary, Boston, Massachusetts and the William Beaumont Hospital, Royal Oak, Michigan, and it conformed to the tenets of the Declaration of Helsinki. Eligible patients were enrolled in this study after they gave informed consent either in person, over the phone, or through the mail, before answering questions to a standardized questionnaire and donating 10 to 50 ml of venous blood.

Details of the recruitment of patients and their siblings are described elsewhere [25,29]. In brief, we recruited 244 individuals comprising 104 extremely discordant sib pairs, all all of Northern European descent and 50 years of age or older. All index patients had the neovascular form of AMD in at least one eye, defined by subretinal hemorrhage, fibrosis, or fluorescein angiographic presence of neovascularization documented at the time of, or before, enrollment in the study. Patients whose only exudative finding was a retinal pigment epithelium detachment were excluded because this finding may not represent definite neovascular AMD and, therefore, the severe phenotype we sought. Also excluded were patients with signs of pathologic myopia, presumed ocular histoplasmosis syndrome, angioid streaks, choroidal rupture, any hereditary retinal diseases other than AMD, and previous laser treatment due to retinal conditions other than AMD.

The unaffected siblings had normal maculae at an age older than that at which the index patient was first diagnosed with neovascular AMD. Maculae were defined as the zone centered at the foveola and extending 2 disc diameters (3000 microns) in radius. Normal maculae fulfilled the following criteria: 0–5 small drusen (all less than 63 microns in diameter), no pigment abnormalities, no geographic atrophy, and no neovascularization (as defined previously [26,29]; AMD “category 1” or less on the AREDS scale). Disease status of every participant was confirmed by at least two of the investigators by evaluation of fundus photographs or fluorescein angiograms except when one of the investigators directly examined an unaffected sibling during a home visit (n=4 cases).

Additionally, we administered a standardized questionnaire to all eligible participants in person or over the phone to ascertain smoking exposure measured in pack years, BMI, and history of diabetes. We used the date of the index patient’s fundus photographs as our cutoff date for smoking exposure for both members in a sibship. In most cases, the diagnosis of AMD was made simultaneously with the diagnosis of neovascular AMD.

### Genotyping

**Figure 1:**
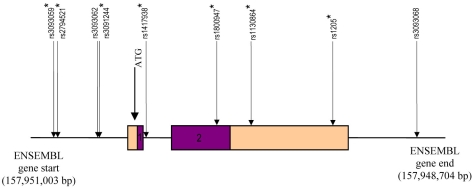
Schematic of the C-reactive protein gene representing the promoter region, the 2 exons, and the 3′ untranslated region. The coding regions of the exons are colored dark. The untranslated regions are depicted in the lighter color. All nine single nucleotide polymorphisms (SNPs) were genotyped by direct sequencing except for rs3093068 which was genotyped using the Sequenom technology. The asterisks represent the 7 SNPs that define common variation in CRP. Please note that 3 of these SNPs are located several base pairs upstream from the ATG start site. Although we sequenced 92.3% of the CRP gene, no novel variation was found.

For all molecular procedures leukocyte DNA was either purified by using standard phenol-chloroform or DNAzol (Invitrogen Corporation, Carlsbad, CA) extraction protocols. Oligonucleotide primers were selected using the Primer3 program to encompass the promoter, both exons, including splice sites and the 3′-UTR of *CRP*. Primer pairs were designed according to the CRP gene sequence in Ensembl and can be seen in Table 1. The fragments analyzed included the set of established common *CRP* SNPs (Figure 1 and Table 2). For all amplicons, polymerase chain reaction (PCR) was used to amplify genomic DNA fragments from 20 ng of leukocyte DNA in a solution of 10× PCR buffer containing 25 mM of MgCl_2_, 0.2 mM each of dATP, dTTP, dGTP, and dCTP, and 0.5 units of Taq DNA polymerase (USB Corporation, Cleveland, OH). Next, 5M betaine was added to each PCR resulting in a final concentration of 1.5M (Sigma-Aldrich, St. Louis, MO). The temperatures used during the polymerase chain reaction were as follows: 95 °C for 5 min followed by 35 cycles of 58 °C for 30 s, 72 °C for 30 s, and 95 °C for 30 s, with a final annealing at 58 °C for 1.5 min and extension of 72 °C for 5 min. For sequencing reactions, PCR products were digested according to manufacturer’s protocol with ExoSAP-IT (USB Corporation) then were subjected to a cycle sequencing reaction using the Big Dye Terminator v3.1 Cycle Sequencing kit (Applied Biosystems, Foster City, CA) according to the manufacturer’s protocol. Products were purified with Performa DTR Ultra 96-well plates (Edge Biosystems, Gaithersburg, MD) to remove excess dye terminators. Samples were sequenced on an ABI Prism 3100 DNA sequencer (Applied Biosystems). Electropherograms generated from the ABI Prism 3100 were analyzed using the Lasergene DNA and protein analysis software (DNAStar, Inc., Madison, WI). Electropherograms were read by two independent evaluators without knowledge of the subject’s disease status. All patient DNAs were sequenced in the forward direction (5′ to 3′), unless variants or polymorphisms were identified, in which case confirmation was obtained in some cases by sequencing on the reverse strand.

**Table 1:** Primers Used

**Location**	**Forward primer**	**Reverse primer**
5′-UTR/promoter	TTTACTGTCAGGGCCGTCAT	TCTCTCAGGGCTCCACTTTG
Exon 1, Intron 1	TCTTCCCGAAGCTCTGACAC	ACACACACCATGAAGGATGC
Exon 2 (part 1)	GTGTAACTGGAGAAGGGGTCA	CTTCTGCCCCCACAGTGTAT
Exon 2 (part 2)	TACAGTGGGTGG GTCTGAAA	TGGGAACCATGCAGTGTAAA
3′-UTR	GCCCTTCAGTCCTAATGTCC	AGATCAGCGCTTCCTTCTCA
3′ UTR	TGGTTTTTG TTTGCTTGCAG	TGGGCAGTCCAGGTGTAGAT

All primers are written in the 5′-3′ direction.

**Table 2:** Results of single marker analysis from the family-based association test, assuming an additive genetic model

**SNP**	**Number of informative families**	**Variance (S)**	**Z value**	**p value**
rs3093059 ^†^	15	3.604	1.010	0.31
rs2794521 ^†^	41	11.408	0.197	0.84
rs3093062	2	*****	*****	*****
rs3091244 ^†^	42	13.580	1.123	0.26
rs1417938 ^†^	46	12.718	0.365	0.72
rs1800947 ^†^	9	3.075	0.855	0.39
rs1130864 ^†^	45	12.546	0.885	0.38
rs1205 ^†^	46	16.403	0.593	0.55
rs3093068 *	12	3.00	0.577	0.564

Results of Family-Based Association Test (FBAT) assuming an additive genetic model. All nine genotyped single nucleotide polymorphisms (SNPs) are shown, including those seven that represent common genetic variation within CRP (as indicated by †). SNPs that had less than 4 informative families (as indicated by *****) could not be analyzed. Results shown are based on 101 families, using one pair per family.

So that appropriate inferences between common variation in CRP and AMD risk could be made, we analyzed the 3′-UTR SNP that was part of the previously reported significantly associated AMD risk haplotype [22] using the Sequenom technology (Sequenom, Inc., San Diego, CA). PCR primers were designed by the Sequenom Spectro Designer software (version 3.0.0.3) by inputting sequence containing the SNP site and 100 bp of flanking sequence on either side of the SNP. Briefly, 10 ng genomic DNA were amplified in a 5 μl reaction containing 1X HotStar Taq PCR buffer (Qiagen), 1.625 mM MgCl_2_, 500 μM each dNTP, 100 nM each PCR primer, 0.5 U HotStar Taq (Qiagen). The reaction was incubated at 94 °C for 15 min followed by 45 cycles of 94 °C for 20 s, 56 °C for 30 s, 72 °C for 1 min, followed by 3 min at 72 °C. Excess dNTPs were then removed from the reaction by incubation with 0.3 U shrimp alkaline phosphatase (USB) at 37 °C for 40 min followed by 5 min at 85 °C to deactivate the enzyme. Single primer extension over the SNP was performed in a final concentration of between 0.625 μM and 1.5 μM for each extension primer (depending on the mass of the probe), iPLEX termination mix (Sequenom) and 1.35 U iPLEX enzyme (Sequenom) and cycled using a two-step 200 short cycles program; 94 °C for 30 s followed by 40 cycles of 94 °C for 5 s, 5 cycles of 52 °C for 5 s, and 80 °C for 5 s, then 72 °C for 3 min. The reaction was then desalted by addition of 6 mg cation exchange Clean Resin (Sequenom) followed by mixing and centrifugation to settle the contents of the tube. The extension product was then spotted onto a 384 well spectroCHIP before being analyzed in the MALDI-TOF mass spectrometer. Data was collected, in real time, using SpectroTYPER Analyzer 3.3.0.15, SpectraAQUIRE 3.3.1.1, and SpectroCALLER 3.3.0.14 (Sequenom). Additionally, to ensure data quality genotypes for each subject were also checked manually. For eight SNPs (rs3093059, rs2794521, rs3093062, rs3091244, rs1417938, rs1800947, rs1130864, and rs1205) genotype data was available for 244 individuals. For SNP rs3093068 genotype data were available for 205 individuals. All individuals were previously genotyped for CFH Y402.

### Statistical analyses

**Figure 2:**
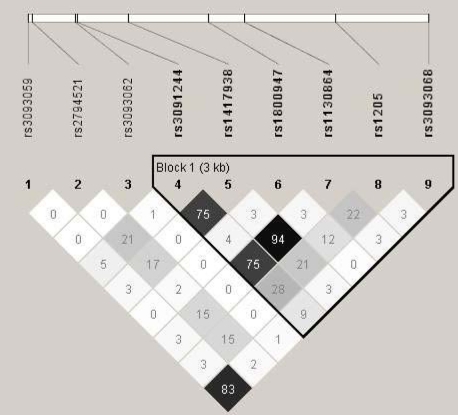
Linkage Disequilibrium of Single Nucleotide Polymorphisms (SNPs) along the 1q25 region encompassing the CRP gene and illustrating the 1 distinct haplotype block, defined by the confidence intervals, an algorithm proposed by Gabriel et al. [31] using HAPLOVIEW. The linkage disequilibrium (r^2^) between any two SNPs is listed in the cross cell. Asterisk means the darker the color indicates the higher the linkage disequilibrium between any two SNPs. Please note that SNP rs3091244 consists of three alleles; two minor alleles A and T are combined into one minor allele due to the limitation of the program which only allows for dichotomous SNPs. SNP rs3093068 was genotyped on 205 subjects, whereas the other eight SNPs were genotyped on 244 subjects.

The program FBAT, which tests for family-based association, was used to evaluate the effect of each SNP individually on risk of AMD [24]. Haploview was used to generate the linkage disequilibrium (LD) plot (Figure 2) among the nine identified SNPs. Linkage disequilibrium (*r*^2^) between each of the nine SNPs is depicted in Figure 2 [30]. The haplotype blocks were constructed by Haploview using the method proposed by Gabriel et al. [31] Individual haplotypes were inferred and tested for association with AMD using FBAT [24]. Conditional logistic regression (CLR; SAS 9.1; SAS Institute Inc, Cary, NC) was performed to identify factors associated with neovascular AMD. Potential risk factors of interest, as defined in the previous section, were evaluated one at a time. For each CRP SNP, the minor allele (in unaffected siblings) in both the homozygous and heterozygous states versus the common allele in the homozygous state was examined in the model (Table 3). Genotype and allele frequencies for all SNPs identified were calculated in the affected and separately in unaffected siblings (Table 4). For this analysis we used one sib pair per family to eliminate the correlation between siblings. Deviation from Hardy–Weinberg equilibrium was tested on each SNP using the χ^2^ test.

**Table 3:** Single factor conditional logistic regression analyses of risk factors for neovascular age-related macular degeneration

**Variable**	**Risk Factor**	**Referent**	**O.R. (95% C.I.)**	**p value**
SNP rs3093059*	CT or CC	TT	0.48 (0.16–1.46)	0.20
SNP rs2794521*	GA or GG	AA	1.02 (0.52–2.00)	0.95
SNP rs3093062	AG or AA	GG	1.23 (0.06–26.00)	0.90
SNP rs3091244*	CT, TT, CA, TA or AA	CC	0.74 (0.35–1.55)	0.42
SNP rs1417938*	TA or TT	AA	0.91 (0.46–1.80)	0. 78
SNP rs1800947*	CG or CC	GG	0.64 (0.17–2.43)	0. 51
SNP rs1130864*	TC or TT	CC	0.72 (0.36–1.45)	0.35
SNP rs1205*	AG or AA	GG	1.24 (0.64–2.42)	0.53
SNP rs3093068**	CC or CG	GG	0.57 (0.17–1.95)	0.37
CFH	CT or CC	TT	2.80 (1.17–6.70)	0.02
CFH	CC	CT or TT	31.62 (4.27–234.07)	0.0007
Smoking	≥10 pk-yrs	<10 pk-yrs	1.97 (1.12–3.46)	0.02
BMI^†^ 20s	≥25	<25	1.31 (0.61–2.81)	0.50
BMI^†^ 30s	≥25	<25	1.37 (0.70–2.70)	0.36
BMI^†^ 40s	≥25	<25	0.89 (0.49–1.64)	0.71
BMI^†^ 50s	≥25	<25	1.44 (0.76–2.75)	0.27
Overall BMI^†^	≥25	<25	0.79 (0.43–1.45)	0.45
BMI^†^ 20s	(20-25)	≤20	1.09 (0.56–2.10)	0.81
BMI^†^ 30s	(20-25)	≤20	1.66 (0.66–4.16)	0.28
BMI^†^ 40s	(20-25)	≤20	3.41 (1.05–11.07)	0.04
BMI^†^ 50s	(20-25)	≤20	1.46 (0.39–5.52)	0.58
Overall BMI^†^	(20-25)	≤20	2.78 (0.86–9.04)	0.09
BMI^†^ 20s	>25	<20	2.87 (0.47–17.5)	0.25
BMI^†^ 30s	>25	<20	N/A	N/A
BMI^†^ 40s	>25	<20	1.00 (0.14–7.10)	1.00
BMI^†^ 50s	>25	<20	N/A	N/A
Overall BMI^†^	>25	<20	N/A	N/A
Diabetes^‡^	Any type of diabetes	No diabetes	0.61 (0.27–1.39)	0.24

Single factor conditional logistic regression analyses of risk factors for neovascular age-related macular degeneration using SAS 9.1. Results from all nine genotyped single nucleotide polymorphisms are shown, including the seven that represent common genetic variation within CRP (as indicated by the *). The variable pk-yrs means pack-years, which was defined as one pack a day for one year. The OR is the odds ratio and C.I. is confidence interval. The variable BMI indicates body mass index, which was calculated by the mean weight in kilograms (excluding pregnancy or lactation) for each decade divided by the square of the height in meters at age 25 years. Decades shown are 20s, 30s, 40s, and 50s. Diabetes status (both insulin and non-insulin dependent), was defined by the regular use of medication as reported by the patient for at least six months before the reference age. N/A means not applicable, the sample size was too small when stratified. Results shown are based on 101 families, using one pair per family.

**Table 4:** Genotype and allele frequencies

**SNP**	**Affected siblings**				
	**Genotype frequency**			**Allele frequency**	
	**Homozygous common**	**Heterozygous**	**Homozygous minor**	**Common**	**Minor**
rs3093059	TT=89.42	CT=9.62	CC=0.92	T=94.23	C=5.77
rs2794521	AA=55.77	GA=36.54	GG=7.69	A=74.04	G=25.96
rs3093062	GG=99.04	AG=0.96	AA=0.00	G=99.52	A=0.48
rs3091244	CC=36.54	TC=47.12	TT=7.69	C=62.98	T=32.21
		AC=5.77	AA=0.96		A=4.81
		AT=1.92			
rs1417938	CC=45.19	TC=47.12	TT=7.69	C=94.71	T=5.29
rs1800947	GG=89.42	CG=10.58	CC=0.00	G=94.70	C=5.30
rs1130864	AA=43.27	TA=49.04	TT=7.69	A=68.75	T=31.25
rs1205	GG=42.31	AG=44.23	AA=13.46	G=64.42	A=35.58
rs3093068	GG=88.97	CG=10.29	CC=0.74	G=94.71	C=5.29

**SNP**	**Unaffected siblings**				
	**Genotype frequency**			**Allele frequency**	
	**Homozygous common**	**Heterozygous**	**Homozygous minor**	**Common**	**Minor**
rs3093059	TT=85.58	CT=14.42	CC=0.00	T=92.79	C=7.21
rs2794521	AA=55.77	GA=38.46	GG=5.77	A=75.00	G=25.00
rs3093062	GG=99.04	AG=0.96	AA=0.00	G=99.52	A=0.48
rs3091244	CC=31.73	TC=46.15	TT=8.65	C=59.13	T=34.13
		AC=8.65	AA=0.00		A=6.73
		AT=4.81			
rs1417938	CC=40.38	TC=50.00	TT=9.62	C=93.75	T=6.25
rs1800947	GG=88.46	CG=10.58	CC=0.96	G=93.75	C=6.25
rs1130864	AA=41.35	TA=49.04	TT=9.62	A=65.38	T=34.62
rs1205	GG=46.15	AG=45.19	AA=8.65	G=68.75	A=31.25
rs3093068	GG=87.22	CG=12.78	CC=0.00	G=93.56	C=6.44

Genotype and allele frequencies of the nine genotyped single nucleotide polymorphisms of the C-reactive protein gene. Genotype and allele frequencies are given for the affected sibs and separately for their unaffected siblings.

## Results

### Demographics of participants

The mean age at enrollment for affected siblings was 71.8 years (range: 49.0–86.5 years). The mean age at enrollment for the unaffected siblings was 76.1 years (range: 50.3–93.9 years). As reported in the methods section, to ascertain epidemiological exposures, we calculated the reference age for both affected and unaffected subjects based on the date of neovascular AMD diagnosis of the affected sibling. Therefore, the mean age of our unaffected siblings at the time of their affected siblings’ diagnosis of neovascular AMD was 72.3 years (range: 41.3–90.9; SD=8.8) for ascertainment of epidemiological exposures. In addition, 40% of the unaffected siblings were male, and 43% of the matching affected cases were male.

We sequenced 92.3% of the *CRP* gene which encompasses 961 bp from the first ATG to the stop codon (TGA) according to Ensembl. Additionally, to ensure we captured the previously reported CRP common variation, we sequenced a 925 bp region of the 5′UTR and 1,202 bp region of the 3′UTR. No new variation was uncovered within any of the CRP fragments analyzed. Aside from the seven SNPs representing common variation, a previously reported SNP (rs3093062) was identified in the promoter region (Table 2, Table 3, and Figure 1) [16-18,21]. We did not find a statistically significant association between any of the nine CRP SNPs representing common and risk of neovascular AMD using the family-based association test (Table 2), single factor CLR (p≥0.2; Table 3) or McNemar’s test (data not shown). When we controlled for smoking exposure (≥ 10 package years or < 10 package years), BMI by decade and over a lifetime, as well as *CFH* genotype, none of the minor alleles demonstrated any significant association with neovascular AMD either (data not shown). Though presence of diabetes and a BMI greater than 25 were both higher in unaffected siblings (36.2% and 42.9% respectively) when compared to affected siblings (12.9% and 20.7% respectively) both single factor CLR and the McNemar’s test showed no significant association between BMI and neovascular AMD, or between diabetes status and neovascular AMD (Table 3).

No significant deviations from Hardy–Weinberg equilibrium (HWE) for any of the genotypes studied in *CRP* was observed in either the affected or unaffected sets of siblings suggesting no contamination of our data set (Table 4). When testing for significant departures from HWE, we used one degree of freedom for the biallelic SNPs (rs3093059, rs279452, rs3093062, rs1417938, rs1800947, rs1130864, and rs1205) and three degrees of freedom for the SNP rs3091244, which has three alleles.

Six SNPs (rs3091244, rs1417938, rs1800947, rs1130864, rs1205, and rs3093068) constituted a haplotype block of which only rs1130864 and rs1417938 were in high LD (*r*^2^=0.94) (Figure 2) [30]. SNP rs3093068 was in LD but less so with rs3093059 (*r*^2^=0.83), which was not part of the haplotype block. SNPs which made up the haplotype block comprised six different haplotypes with ≥ 5% frequency (Table 5, Table 6, Table 7). Haplotype analysis using the family-based association approach showed no significant association between any of the six haplotypes and AMD (Table 5 and Table 7). In an effort to replicate the findings by the Rotterdam Study [22] we also conducted haplotype analysis on the three SNPs (rs1130864, rs1205, and rs3093068) analyzed in that study that defined common variation and were part of the same haplotype block (Figure 2 and Table 7). Although the frequencies for the four haplotypes in our population (h1: 0.33, h2:0.32, h3:0.30, and h4:0.06) were similar to those in the Rotterdam population (h1: 0.33, h2:0.32, h3:0.28, and h4:0.05) we did not find a statistically significant association with AMD risk (Table 6). When we stratified the haplotypes according to CFH genotype, we were only left with a handful of sib pairs in each subgroup (6–12) and were thus relatively underpowered to detect an association in this manner.

**Table 5:** Haplotype analysis

**Order**	rs3091244	rs1417938	rs1800947	rs1130864	rs1205	rs3093068	**Number of Informative Families**	**Frequency**	p value*
h1	C	A	G	C	G	G	31.1	0.335	0.9214
h2	T	T	G	T	G	G	31.6	0.272	0.9233
h3	C	A	G	C	A	G	31.6	0.254	0.494

Haplotype analysis of the C-reactive protein gene using the program FBAT. The single nucleotide polymorphisms used in this analysis are based on the haplotype block produced by Haploview and shown in Figure 2. Haplotypes shown are only those that occurred at a frequency of greater than 5% in our population. The p-values given are based on 100,000 permutations.

**Table 6:** Full Haplotype Analysis

**Order**	rs3091244	rs1417938	rs1800947	rs1130864	rs1205	rs3093068	**Frequency**
h1	C	A	G	C	G	G	0.335
h2	T	T	G	T	G	G	0.272
h3	C	A	G	C	A	G	0.254
h4	A	A	G	C	G	C	0.254
h5	C	A	C	C	A	G	0.054
h6	C	T	G	C	A	G	0.051
h7	T	A	G	T	G	G	0.004
h8	A	A	G	C	A	C	0.003
h9	C	A	G	T	G	G	0.003
h10	T	T	G	C	G	G	0.003
h11	C	T	G	T	G	G	0.003
h12	T	A	G	C	G	C	0.003
h13	A	A	C	C	A	G	0.003
h14	T	A	C	C	A	G	0.003
h15	T	T	C	T	A	G	0.003
h16	T	T	G	T	A	G	0.001

All haplotypes produced by the FBAT analysis of the C-reactive protein gene. The frequency of these haplotypes in our population is shown. Please note that many of these haplotypes occurred infrequently in our population.

**Table 7:** Haplotype comparison

**Despriet et al. 2006 [22]**				
**Order**	rs1130864	rs1205 *	rs3093068	**Frequency**
h1	C	T	C	0.33
h2	T	C	C	0.32
h3	C	C	C	0.3
h4	C	C	G	0.05

**Current study**				
**Order**	rs1130864	rs1205	rs3093068	**Frequency**
h1	C	A	C	0.33
h2	T	G	C	0.32
h3	C	G	C	0.28
h4	C	G	G	0.06

Abbreviations: h1, haplotype 1. *NOTE: Despriet et al. sequenced rs1205 in the reverse direction, resulting in the complement alleles. Replication of the Rotterdam Study [22]. The first haplotype results shown are from the Rotterdam study, using the three Single Nucleotide Polymorphisms rs1130864, rs1205, and rs3093068 that defined common variation and were part of the same haplotype block (Figure 2 and Table 6). The second haplotype results are from our population. Please note that the Rotterdam Study sequenced rs1205 in reverse, resulting in the complement alleles.

## Discussion

In summary, no statistically significant association was detected between any of the nine SNPs identified in the *CRP* gene and neovascular AMD when considering disease status alone or with stratification by smoking exposure, BMI, or *CFH* genotype. Haplotype analysis resulted in the same findings as single factor SNP analysis, demonstrating that there was no association between the *CRP* gene and risk of neovascular AMD. Our findings are supported by similar results from the Physician’s Health Study that showed no association between common variation in the *CRP* gene and risk of AMD after controlling for *CFH* genotype [23]. Additionally, direct sequencing of *CRP* in our extremely discordant sib pair population uncovered no new variation. These findings taken together could suggest that if elevated circulating levels of CRP are associated with AMD, it is likely not due to genetic variation within *CRP* but likely variation in some other gene or epidemiological risk factor. Further supporting this hypothesis is that in a study of over 3,000 subjects [32] variation in circulating *CRP* levels was accounted for by phenotypic factors (such as a high BMI > 25) rather than *CRP* genotype (26% versus 1.4%). Nevertheless, analysis of three common variants in the 3′-UTR within *CRP*, on a prospective study population from the Netherlands [22] showed that haplotypes associated with higher circulating *CRP* levels are protective in individuals who are *CFH* Y402, and these same haplotypes can confer increased risk on AMD in individuals who are *CFH* 402H. It is important to note that in the Rotterdam study, the haplotypes studied were not directly associated with AMD risk [22]. Although our population of neovascular AMD (n=116) was slightly higher than that of the Rotterdam study (n=78), which did not differentiate between the advanced subtypes of AMD, it may be that our results are inconclusive as we were relatively underpowered when we stratified subjects according to *CFH* genotype. Since subjects in both of these studies are Caucasian, it could also be that if variation in *CRP* increases susceptibility to advanced AMD, it may predispose to only the atrophic subtype. Another possibility is that *CRP* variants may have a small or modest influence on AMD risk, or there may exist multiple susceptibility genes for AMD that are not necessarily expressed in every patient.

To assess the power of the current study, we used a power calculation specifically designed for discordant sib pair studies, given the genetic parameters. We made the following reasonable assumptions that we felt reflected our knowledge on the *CRP* gene: 1) the *CRP* gene is a weak AMD risk factor with relative risk from 1.5 to 3; 2) the prevalence of AMD in people older than 60 is 0.2 [32]; 3) the significance level is 0.01, which considers multiple testing correction without overcorrecting; and 4) the genetic proportion (the percentage of cases with AMD that is due to disease genotype) ranges from 0.3 to 0.6. The current data contains 104 sib pairs for the majority of the SNPs analyzed. Both dominant and recessive genetic models are considered. The power of the study ranges from 7% to 78% under a dominant model and ranges from 4% to 60% under a recessive model. In both dominant and recessive models, the power is low when the relative risk and genetic proportion are low; the power increases when relative risk and genetic proportion increase. When the relative risk is 1.5, the power is very low, around 5%, there is little or no power to detect any association; when the relative risk approaches 3 and the genetic proportion is close to 50% to 60%, the study possesses a power > 70% to detect the association.

In summary, our analyses of extremely discordant sib pairs suggest that it is unlikely that genetic variants in CRP are involved in the pathogenesis of AMD, and particularly neovascular AMD. The correlation between plasma levels of *CRP* and AMD risk observed in some cohorts may serve as a general indicator of the role of inflammation in AMD, but does not appear to provide specific insights regarding molecular mechanisms contributing to disease.
